# Development and psychometric properties of the parent version of the Profile of Neuropsychiatric Symptoms (PONS) in children and adolescents

**DOI:** 10.1186/s12887-015-0376-x

**Published:** 2015-05-19

**Authors:** Paramala Santosh, Paul Gringras, Gillian Baird, Federico Fiori, Regina Sala

**Affiliations:** Department of Child and Adolescent Psychiatry, Institute of Psychiatry, Psychology & Neuroscience, King’s College London, PO Box 86, 16 De Crespigny Park, London, SE5 8AF UK; Children’s Sleep Medicine Department, Paediatric Neurosciences, Evelina London Children’s Hospital, St Thomas Hospital, London, UK; Paediatric Neurosciences, Evelina London Children’s Hospital, St Thomas Hospital, London, UK

**Keywords:** Profile Of Neuropsychiatric Symptoms scale, Patient reported outcomes, Patient centred outcome measures, Internet-based scale, Psychometric properties, HealthTracker™

## Abstract

**Background:**

The use of neuropsychiatric Patient Centred Outcome Measures (PCOMs) in routine child mental health and paediatric services is very time consuming and often requires multiple scales being completed as no single scale covers all areas of psychopathology. The use of a web-based programme can overcome these problems and contribute to improved use of PCOMs in clinical practice. We aim to develop a web-based scale (using HealthTracker™) to screen and identify young people with significant neuropsychiatric symptoms to enable early intervention.

**Methods:**

Qualitative development of the Profile of Neuropsychiatric Symptoms (PONS) and quantitative evaluation of the psychometric properties of the PONS scale (parent version). Parents of 929 from the general population and 147 with neuropsychiatric disorders (5–18 years old) completed the PONS online. In addition, those children with neuropsychiatric disorders were assessed for the presence of current and lifetime psychiatric disorders using the Development and Well-Being Assessment (DAWBA).

**Results:**

The PONS scale (parent version) consists of 30 symptom domains rated on a 7-point scale for both frequency and impairment. We found an intra-class correlation coefficient for single measures was 0.44 (0.42-0.46 95 % CI, F = 22.84, p ≤ 0.0001) and for average measures was 0.96 (0.95-0.96 95 % CI, F = 22.84, p ≤ 0.0001). The factor analysis showed a 4-factor model: Neurodevelopmental Disability; Behavioural and Emotional Dysregulation; Psychoses and Personality Dysfunction; and Anxiety and Depression. The receiver operating characteristic area for the 4-factors was 0.96 (SE = 0.006; 0.95-0.97 95 % CI).

**Conclusions:**

The PONS scale (parent version) is a web-based PCOM on the HealthTracker™ system that is a rapid, engaging measure that has excellent reliability and validity. The system allows for automated scoring and immediate feedback of statistical cut-off points and assists clinicians with diagnostic decision-making and optimises use of clinician time.

**Electronic supplementary material:**

The online version of this article (doi:10.1186/s12887-015-0376-x) contains supplementary material, which is available to authorized users.

## Background

Epidemiological research has shown that between 12 % and 14 % of young people have a mental disorder causing significant functional impairment [1, 2], 5 % have neurodevelopmental disorders [3]. However, only about one quarter of them are in contact with specialist mental health services [4, 5].

Methods for assessing the prevalence and community burden of psychiatric disorders in children and adolescents have improved dramatically in the past decade [1]. There is a broad range of standardized structured interviews and self-report assessments that generate Diagnostic and Statistical Manual (DSM) Mental Disorder diagnoses with good reliability and validity [1]. Using neuropsychiatric Patient Centred Outcome Measures (PCOMs) in routine child mental health and paediatric services is very time consuming and often requires multiple scales being completed as no single scale covers all areas of psychopathology. Most children and adolescents attending mental health or neurodisability services have symptoms of more than one emotional or behavioural developmental disorder. Currently, there are many disorder-specific rating scales, but none that are child or parent rated that include symptoms of neurodevelopmental disorders such as attention deficit hyperactivity disorder and autism spectrum disorders alongside symptoms of psychoses, bipolar disorder, anxiety and depression. The commonly used measures such as the Child Behaviour Check List (CBCL) [6], and the Strength and Difficulties Questionnaire (SDQ) [7] currently do not cover all the above areas adequately. There is an urgent need to develop brief PCOMs for childhood neuropsychiatric disorders and neurodisability, that can be completed in around 10 min, capture frequency and impairment arising from symptoms of multiple, relevant, co-occurring disorders in a dimensional manner allowing its use across conditions. It would be useful if the PCOM could be used longitudinally to capture change. There is a need to capture domain-specific, dimensional frequency and impairment ratings, which would uniquely allow the PCOM to be used across diagnostic categories, allowing for use in research using the NIH Research Domain Criteria (RDoC) [8] (http://www.nimh.nih.gov/research-priorities/rdoc/index.shtml). The RDoC project intends to develop, for research purposes, new ways of classifying mental disorders based on behavioral dimensions and neurobiological measures [8]. RDoC attempts to bring the power of modern research approaches in genetics, neuroscience, and behavioral science to the problems of mental illness, studied independently from the classification systems by which patients are currently grouped.

PCOMs include the self/proxy assessments of symptoms, functional status, or other concerns such as patient needs and satisfaction with care ([9–15]. The use of a web-based programme can contribute to improved use of PCOMs in clinical practice [16]. Technology can enhance the lives of most individuals, especially those with neurodisabilities [17–20], who will benefit through the use of the HealthTracker™, an existing web-based health-monitoring platform. HealthTracker™ is an established platform for online collection and storage of medical data that allows multi-modal presentation of questionnaires (including animated scale presentations for young children) and assists in automatically allocating questionnaires based on developmental level rather than chronological age. Online display allows questionnaires to be presented in a client-friendly manner in many languages, assisted by audio recordings for those who have dyslexia and visual impairment [21, 22]. In addition, being web-based, it allows for more frequent assessment without clinic visits.

Children and adolescents with intellectual disabilities and challenging behaviours can make conventional assessment difficult [23]. For the reasons stated above, more work is needed to improve PCOMs, especially harnessing technology, to screen young people and identify early childhood onset of psychiatric disorders to enable early intervention; especially in those with neurodisability. The Profile Of Neuropsychiatric Symptoms (PONS) was developed to screen and capture information on specific symptom domains that are commonly seen in neurodevelopmental, neuropsychiatric and emotional and behavioural disorders in children and young people, developed in a manner to capture change in symptom frequency and impairment across time, using current FDA protocols for development of PCOMs and the NIH RDoC agenda. It was developed with a view to capture symptoms of developmental disorders such as ADHD, ASD, OCD, motor coordination disorder; disruptive disorders such as ODD and Conduct Disorder; Psychoses, Bipolar Disorder and Emerging Personality Disorder; and Anxiety and Depressive Disorders. Currently no existing single scale is able to capture all these symptoms in a scale that can be completed in around 10–12 min, which can also be used to track change between clinic visits.

The objective of this project was to develop the web-based PONS. For this purpose, we followed the FDA recommendations for patient reported outcome measures (currently called PCOMs) [24, 25] and principles used in the Patient Reported Outcomes Measurement Information System (PROMIS). PROMIS is an NIH-funded initiative to develop instruments to be used across chronic conditions as a system of highly reliable, precise measures of patient–reported health status for physical, mental, and social well–being (www.nihpromis.org).

The Profile of Neuropsychiatric Symptoms (PONS) consists of three child versions - for young children (5–7 years old), children (8–11 years old) and adolescents (12–18 years old); a parent or carer version, a teacher version, and a clinician version. This paper will report on the parent version of the PONS and its qualitative development, and the quantitative evaluation of its psychometric properties in child and adolescent controls from the general population and a group with neuropsychiatric disorders.

## Methods

### Phase 1: qualitative development of the PONS scale

Institutional Review Board approval was obtained from the Guy’s and St Thomas’s Hospitals NHS Trust before subject enrolment. Online informed consent was obtained from all control participants (i.e., child, adolescent, parent) before study instruments were administered. In the clinic sample, the PONS was completed as part of routine clinical care and we had consent for anonymized use of all clinical data.

The uniqueness of this instrument is that each domain describes what a child with a specific symptom domain would look like, rather than focusing on individual items, for example, hyperactivity is described as ‘a child who is unable to sit still, is constantly on the go, fidgets, is restless.’ This allows the parent and child to understand what constitutes hyperactivity rather than having separate symptom items to capture the domain of hyperactivity. Initial symptom domains of the PONS scale was based on a detailed literature review, followed by a consensus of child and adolescent psychiatrists and neurodevelopmental paediatricians with extensive experience in neuropsychiatric disorders of young people, and families with children with neuropsychiatric problems. The initial draft of the PONS scale was discussed with a panel of experts of child psychiatrists, paediatricians, neurodevelopmental paediatricians, psychologists, and occupational therapists - each of whom had a minimum of 5 years experience working with children in paediatric or child mental health settings. Each of them was asked to rate the importance and relevance of the various domains for the purpose of the PONS. There was 100 percent agreement regarding the merits of inclusion of the domain in the draft version of the PONS and a consensus reached about its relevance. Redundant or overlapping symptom domains were removed. The second draft of the PONS scale was then presented to young people (aged 5 to 18 years), and their parents for feedback, and to obtain information about comprehensibility and appropriateness of both the content and format of the questions. Focus groups for young people and their parents were conducted separately to understand their different perspectives. The mean duration of these focus groups was 90 min for young people and their parents. The major topics evaluated in the focus groups were structure (e.g., format of the questions, response options), content (e.g., meaning of each symptom domain, language used to describe it), and other aspects such as frequency of administration and recall period. Finally, a discussion between experts in child and adolescent neuropsychiatry and paediatric neurodisability was held based on the summary reports by the focus groups and the final version of the PONS scale was developed for young people and parents, as well as for clinicians. It was ensured that the PONS covered child or parent rated symptoms of neurodevelopmental disorders such as attention deficit hyperactivity disorder and autism spectrum disorders, alongside symptoms of psychoses, bipolar disorder, anxiety and depression. The final PONS scale (parent version) consisted of 30 domains with response options on a 7-point Likert scale because developmentally up to 7-point options can be easily distinguished by adults [26, 27] and feedback from focus groups suggested it to be best option for the PONS. Also, having 7-point Likert scale allows a greater likelihood of being able to capture subtle change accurately across time. The PONS scales are incorporated into the HealthTracker™ platform [21] and are used in different clinical and research settings. Currently, the HealthTracker™ system is used in two EU FP7 projects - the Suicidality: Treatment Occurring in Paediatrics (STOP study; www.stop-study.com) [22] and the Managing the Link and Strengthening Transition from Child to Adult Mental Health Care (MILESTONE) project (www.milestone-transitionstudy.eu).

### Phase 2: psychometric evaluation of the PONS scale

#### Subjects and procedures

After Institutional Review Board approval we used two samples in order to examine the psychometric properties of the PONS scale: a) a convenience sample of 929 children and adolescents from the general population (466 boys and 463 girls); children (5–12 years old; mean age 10.1 years, SD = 3.99) and adolescents (12–18 years old; mean age 13.9 years, SD = 3.65). Professional online marketing resources helped identify ideal user groups to target (such as parenting websites). Online guidance was clear that our target audience was parents of primary and secondary school children without a previous history of behavioural and emotional problems, or involvement with child mental health services.

b) 147 children and adolescents (109 boys, 38 girls), aged 5 to 18 years (mean = 11.09, SD = 3.2) with neuropsychiatric disorders, recruited from the Centre for Interventional Paediatric Psychopharmacology (CIPP) at the Maudsley Hospital, London. Parents were interviewed at intake about the neuropsychiatric symptoms of their children using the PONS (parent version). In addition, for the neuropsychiatric condition sample, children and parents were also interviewed for the presence of current and lifetime psychiatric disorders and completed the Development and Well-Being Assessment (DAWBA) [7].

The data capture, storage, and e-monitoring was conducted online using the HealthTracker™ system. HealthTracker™ is an established health-monitoring platform for online collection, storage of medical data, and data-export to an SPSS database.

#### Statistical analyses

SPSS version 20.0 was used for the analyses. Descriptive statistics was used to characterize both samples. Reliability was assessed using Cronbach’s alpha, alpha if deleted analysis, intra-class correlation, and factor analysis using the general population. Exploratory factor analysis with Promax rotation and Kaiser normalization was done with rotation converged in 10 interactions. Principal axis factoring without fixed number of factors was used. Promax rotation was used with a maximum interaction for convergence of 0.25 (Kappa = 4). A priori threshold to determine the loading of factors was set. Given the explorative purpose of the present study and our sample size we set the factors’ loading threshold at >0.25 [28–33]. Only the greater value was considered when cross-loadings presented a gap >0.2 between the loadings. In addition, Kaiser-Meyer-Olikin (KMO) measure of sampling adequacy and Bartlett’s test of sphericity was provided.

Validity was assessed using the receiver operating characteristic (ROC) analyses. This was computed to evaluate the discriminative power of the factors between both samples. This has been detailed in Additional file 1 (details available in the Results section). In addition, independent samples t-tests were performed with grouping variable DAWBA diagnoses (coded in 1 for positive and 0 for negative diagnosis) and single factor’s scores as dependent variables to assess the PONS ability to distinguish between different groups of patients and to classify them on the basis of DAWBA diagnosis.

PONS’ cut off scores, sensitivity, and specificity was also computed. All p-values are for two-tailed tests with α = 0.05.

## Results

### Phase 1: qualitative development of the PONS scale

Parents specifically supported the strategy of the PONS consisting of domains that describe specific areas of dysfunction and believed that the traditional approach of having many separate items for each domain would lead to a longer scale and reduce uptake. The final PONS scale (parent) consisted of 30 symptom domains. Each domain includes its name domain, its description, and two questions, one about the frequency and the other one about impairment. The HealthTracker™ system ensures that the impairment question only appears if the frequency question is answered as being present, thus speeding up completions. This feature was strongly supported by all the users during the focus groups. All symptom domains are rated on a 7-point scale and the recall period was 1 month for baseline administration.

### Phase 2: psychometric evaluation of the PONS scale

#### Subjects

The sample with neuropsychiatric conditions consisted of 147 children and adolescents with various psychiatric disorders, some of who had multiple comorbid disorders. 75.5 % had Attention Deficit Hyperactivity Disorder (ADHD) (n = 111), 65.3 % had Autism Spectrum Disorder (ASD) (n = 96), 40.1 % had Oppositional Defiant Disorder (ODD) or Conduct Disorder (n = 59), 21.1 % had psychoses or Bipolar Disorder (BP) (n = 31), 54.4 % had Anxiety or Depressive Disorder (n = 80), 25.9 % had Developmental Coordination Disorder (n = 38), and 24.4 % had Obsessive Compulsive Disorder and/or tics (n = 36).

### Reliability

#### Factor analysis

As shown in Table 1, factor analysis was completed on the 30 symptom domains. A 4-factor model was determined to best fit the data based on the screen plot. Based on the pattern of symptom domain loading, the 4-factors were named: (1) Neurodevelopmental Disability (predominantly ADHD, ASD), (2) Behavioural and Emotional Dysregulation (ODD, CD), (3) Psychoses and Personality Dysfunction (Psychoses, BP, emerging PD, spontaneous abnormal movements [34]), (4) Anxiety and Depression (Anxiety and Depressive Disorders). These factors capture the bulk of children and adolescents with mental health problems and intuitively follow clinical and diagnostic clusters.

**Table 1:** Factor Analyses of the Profile of Neuropsychiatric Symptoms (PONS) parent-version using database of general population

Domains	Factors			
	1 : Neurodevelopmental Disability	2 : Behaviour and Emotional Dysregulation	3 : Psychoses and Personality Dysfunction	4 : Anxiety and Depression
Language problems	.670	.066	.053	.049
Clumsiness	.662	−.121	.111	.123
Difficulties learning	.661	.003	−.058	.089
Social communication difficulties	.617	.010	.041	.195
Inattention	.614	.360	−.177	−.012
Mannerisms	.566	−.070	.476	−.136
Impulsivity	.548	.457	−.074	−.111
Hyperactivity	.538	.351	.083	−.198
Cognitive rigidity	.524	.163	−.045	.214
Sensory symptoms	.501	−.185	.127	.298
Circumscribed interests	.398	.046	.192	.127
Obsessions compulsions	.385	−.069	.266	.241
Body control	.348	.023	.286	−.191
Aggression	−.035	.862	.018	.011
Oppositionality	.098	.803	−.083	−.007
Explosive rage	.010	.734	.026	.083
Lack remorse	.157	.552	.076	.062
Labile mood	.051	.533	.011	.271
Eating problems	.166	.210	.037	.193
Hallucinations	.023	−.116	.702	.058
Spontaneous abnormal movements	.236	−.069	.668	−.133
SelfInjury	.021	.129	.556	.081
Antisocial behaviour	−.132	.423	.524	−.046
Paranoid thoughts	−.060	.158	.375	.337
Manic symptoms	.190	.129	.347	.117
Worries	.177	.032	−.129	.709
Low mood	−.048	.150	.004	.685
Fears	.291	−.089	−.051	.589
Depressive thoughts	−.223	.239	.317	.505
Sleep problems	.255	.174	−.043	.285

Extract Method: Principal Axis Factoring. Rotation Method: Promax with Kaiser Normalization. Rotation converged in 10 interactions

The KMO was 0.97 (X^2^ = 20,507, 54) with a Bartlett’s test of sphericity of 378, p ≤ 0.001.

#### Internal consistency

Cronbach’s alpha was 0.96 for the 30 PONS symptom domains (referred to as items here). There were no negative inter-item correlations and each item-total correlations were above the threshold of >0.20. In addition, the alpha if deleted analysis showed that the Cronbach’s alpha value reduced if any of the PONS items were dropped, meaning that there was no need to drop any items.

#### Intra-class correlation

Intra-class correlation coefficient for single measures was 0.44 (0.42-0.46 95 % CI, F = 22.84, p ≤ 0.001) and for average measures was 0.96 (0.95-0.96 95 % CI, F = 22.84, p ≤ 0.001).

### Validity

#### Receiver Operating Characteristic (ROC) analysis

As shown in Table 2, the ROC area for the 4-factors was 0.96 (SE = 0.006; 0.95-0.97 95 % CI). Results of the ROC analysis for each of the factors are showed in Table 2.

**Table 2:** Receiver operating characteristics of the factor analyses using the Profile of Neuropsychiatric Symptoms (PONS) between general population and clinical sample

	PONS cut off scores	Sensitivity	Specificity	ROC Area	SE	Asymptotic normal	
		(%)	(%)			(95 % CI)	
**Total**	≥77.50	91.80	90.10	.960	.006	.950	.971
**Neurodevelopmental Disability**	≥37.50	91.80	91.10	.961	.006	.949	.973
**Behaviour and Emotional Dysregulation**	≥19.50	87.10	87.40	.936	.009	.918	.954
**Psychoses and Personality Dysfunction**	≥4.54	84.40	83.30	.901	.014	.873	.929
**Anxiety and Depression**	≥11.68	83.00	82.50	.909	.011	.887	.931

ROC: Receiver Operating Characteristics; SE: Standard Error; CI: Confidential Interval

#### T-test analysis

The results showed that the Neurodevelopmental Disability score was significantly higher for patients who presented with an ASD and/or ADHD diagnoses (t_[145]_ = 3.999, p < .001). The ODD and/or CD patients had a statistically significant difference in the Behavioural and Emotional Dysregulation score (t_[145]_ = 3.352, p = .001). Moreover, the Psychoses and Personality Dysfunction score was significantly greater for those individuals who had psychoses and/or bipolar disturbance (t_[145]_ = 2.384, p = .0022). Finally, patients with depression and/or anxiety had significantly greater Anxiety and Depression score (t_[145]_ = 3.045, p = .003). Taken together, these results suggest that the PONS can be used as a guide for clinical categorization.

## Discussion

The PONS scale (parent version) is a web-based PCOM that consists of 30 symptom domains rated on a 7-point scale for both frequency and impairment and takes around 10 min to complete online. The PONS ensures that child and parent rated symptoms of neurodevelopmental disorders such as attention deficit hyperactivity disorder and autism spectrum disorders alongside symptoms of psychoses; bipolar disorder, emerging personality disorders; anxiety and depression exist in a single scale which is brief and easy to use. Results show that the PONS scale is a reliable and valid instrument in screening for psychiatric disorders in children and adolescents with neuropsychiatric conditions.

Specifically, we found an internal consistency value very close to 1.0 suggesting excellent reliability and providing evidence that the symptom domains measure a common underlying construct. In addition, we found medium correlation for individual symptom domains and high correlations for the full instrument, suggesting that each symptom domain is unique.

The PONS factor structure matches the domain framework of the PROMIS (www.nihpromis.org). The 4 PONS factors, (1) Neurodevelopmental Disability, (2) Behavioural and Emotional Dysregulation (3) Psychoses and Personality Dysfunction, and (4) Anxiety and Depression align to an extent to the PROMIS Peer Relationship domain, the PROMIS Anger domain, and the PROMIS Paediatric Anxiety and Depression domains. The PROMIS currently does not have an equivalent for Psychoses and Personality Dysfunction in children and does not capture all symptoms (www.nihpromis.org). The PONS uniquely captures symptoms of developmental disorders such as ADHD, Autism Spectrum Disorder, Obsessive Compulsive Disorder, motor coordination disorder, disruptive disorders such as ODD and Conduct Disorder; Psychoses, Bipolar Disorder, and Emerging Personality Disorder; and Depression and Anxiety Disorders. Currently no existing single scale is able to capture all these symptoms in around 10 min. Whilst the Strengths and Difficulties Questionnaires (SDQ) [7], is a brief, valid and widely used questionnaire, it does not cover psychoses, personality dysfunction, bipolar disorder, etc., and has not been used regularly to capture change between clinic appointments. The Child Behavior Checklist (CBCL) [6] is much longer, more time consuming and is not a simple PCOM that can be completed in around 10 min and does not have the online optimization available for the PONS using audio recordings or animations for the child versions of the PONS. The PONS is unique in that this is an instrument that has been clearly validated for web-based use. The capture of domain-specific, dimensional frequency and impairment ratings, uniquely allows the PONS to be used across diagnostic categories, allowing for use in research using the NIH Research Domain Criteria (RDoC) [8]. Most importantly, the parents were very pleased with the format of the PONS and it is a PCOM that has been developed with the full input of the users.

Optimized, web-based PCOMs using intelligent branching, audio-assistance for those with reading difficulties, adaptations for visual impairment (with large font-size adaptations), availability in multiple languages increases the likelihood of the PCOM being used. All these have been specifically achieved with the PONS on the HealthTracker™. It is likely to increase the use and completions of questionnaires, improve access to care in geographically isolated populations, could be used for automated triaging, and automated, real-time scoring allows for the results to be available for use by clinicians during clinic visits, even if the PONS was completed in the waiting area whilst waiting to see the clinician.

Feedback from parents was very positive from the focus group. Intelligent branching through online delivery from the HealthTracker™ system minimises completion time (maximum 12 min). The HealthTracker™ system also automatically randomises the order of the symptom domains being presented, and thus significantly reduces practice effects.

The HealthTracker™ system has been specifically tested for user acceptance and optimised for the use of children, adolescents and parents, allowing them to complete it effortlessly. The scale also can be read by a recorded voice, which is especially helpful when it comes to those with dyslexia. In addition, the allocation of the versions of the PONS is automatically assigned based on developmental age, rather than chronological age, which is of utmost importance for those with neurodisability.

Furthermore, the HealthTracker™ system allows the PONS to be used for neuropsychiatric screening of children and adolescents and automated triaging in busy clinics. Clinically, the HealthTracker™ platform allows the PONS scores to be automatically calculated and presented in a graphical manner. This real-time shared feedback allows optimal use of face-to-face and non face-to-face clinical time.

The potential limitations of this study include that PONS was primarily developed as a screening measure and to assist in triaging. We have subsequently used the instrument in epidemiological studies and tested its usefulness against other measures. As this instrument is for online use, it was important that normative data be acquired online. This means, however, that we were not able to practically supervise every online entry, and cannot be entirely sure that completions for the normative sample were by parents with children that had no psychiatric problems. We believe that the large numbers recruited means that such errors will be small, and the advantages of test administration in its intended environment outweigh any disadvantages. In addition, the clinical sample is modest in size and comes from a national specialist child and adolescent mental health service (CAMHS) setting and may not be representative of symptom profiles in less severely ill subjects seen in community CAMHS.

However, this neuropsychiatric sample shows that the PONS screened and identified psychiatric conditions with excellent reliability. There is a need to test diagnostic accuracy of the empirically derived PONS diagnoses in children and adolescents in community child and adolescent mental health and neurodisability services and to assess triaging utility. Future longitudinal studies will report the ability of the PONS to capture change in symptom severity and impairment.

## Conclusion

In summary, the PONS scale (parent version) is a HealthTracker™ system-based fast, engaging PCOM with excellent psychometric properties that reports on the frequency and impairment produced by neuropsychiatric symptoms and neurodisability. The PONS-parent version consists of 30 symptom domains, rated on a 7-point scale for frequency and impairment, covers a comprehensive variety of psychopathology - ADHD, ASD, ODD, OCD, Anxiety, Depression, Psychoses and Bipolar Disorder and is especially appropriate for use in those with neurodisabilities. It allows clinicians to profile children and adolescents with emotional and behavioural problems, and could be useful in clinical screening and triaging, and can also be used in clinical trials and epidemiological research. As it is available online with automated scoring and immediate feedback of statistically significant cut-off points, it assists clinicians with diagnostic decision making and optimises use of clinician time. Future research will need to examine PONS as a change measure.

## Additional file

Additional file 1:
**ROC Curves.** ROC Curves for the factor scores and total score of the PONS scale. In the plots, the horizontal axis is the specificity and the vertical axis is the sensitivity.

## Abbreviations

PONS: Profile Of Neuropsychiatric Symptoms
PCOMs: Patient Centred Outcome Measures
PROs: Patient Reported Outcomes
STOP: Suicidality: Treatment Occurring in Paediatrics
DAWBA: Development And Well-Being Assessment
