# Supplementary material for: Development and psychometric properties of the parent version of the Profile of Neuropsychiatric Symptoms (PONS) in children and adolescents
Source: BMC Pediatr. 2015 May 19;15:62. doi: 10.1186/s12887-015-0376-x (PMC4443664; doi:10.1186/s12887-015-0376-x)
Supplement: Additional file 1: — ROC Curves. ROC Curves for the factor scores and total score of the PONS scale. In the plots, the horizontal axis is the specificity and the vertical axis is the sensitivity. [file 12887_2015_376_MOESM1_ESM.pdf]

APPENDIX 1

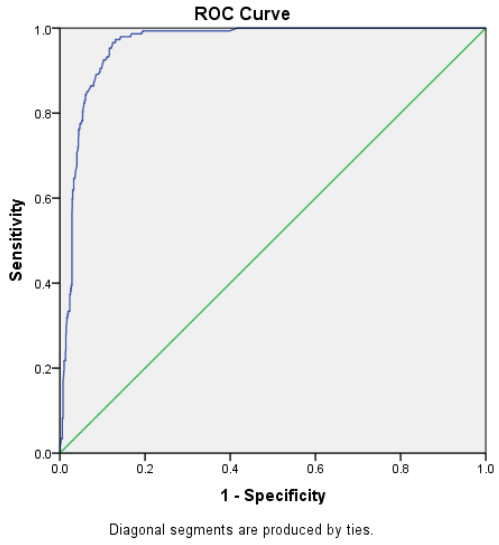

Figure 1: ROC Curve of PONS Total Score

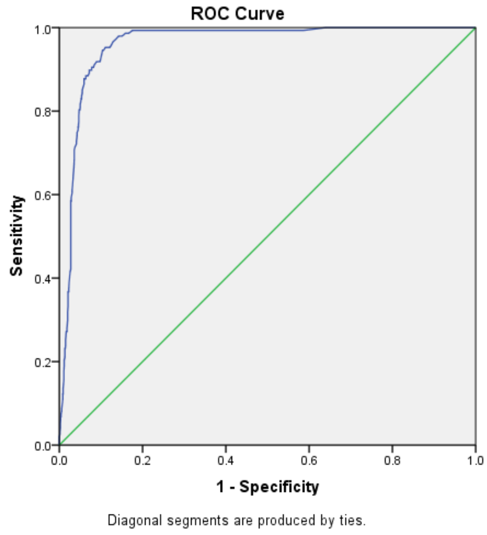

Figure 2: ROC Curve of Factor 1 (Neurodevelopmental Disability) Score

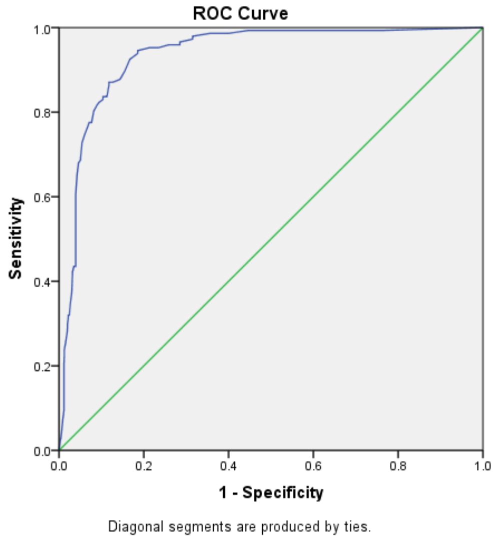

Figure 3: ROC Curve of Factor 2 (Behavioural and Emotional Dysregulation) Score

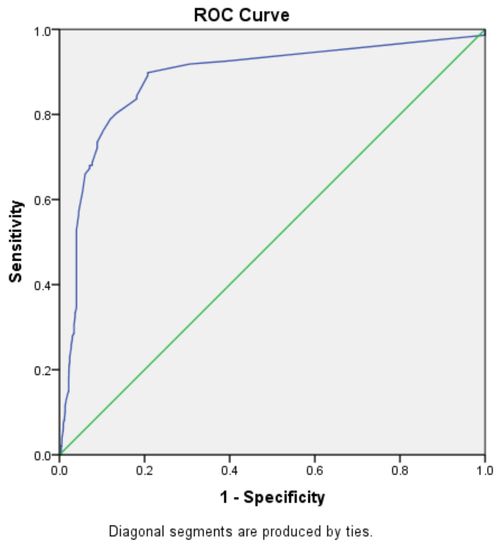

Figure 4: ROC Curve of Factor 3 (Psychoses and Personality Dysfunction) Score

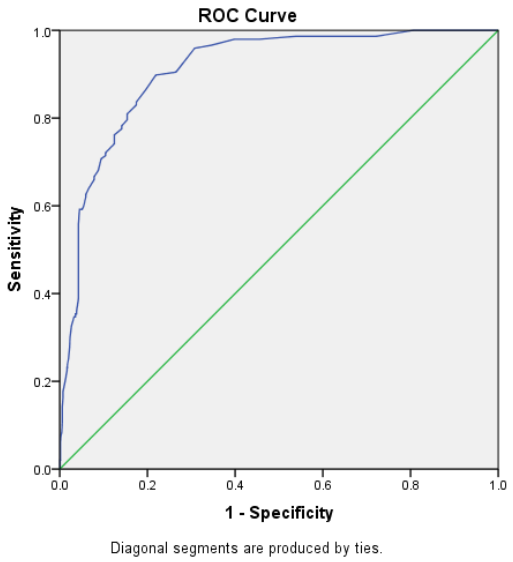

Figure 5: ROC Curve of Factor 4 (Anxiety and Depression) Score
